# Non-Destructive Determination of Starch Gelatinization, Head Rice Yield, and Aroma Components in Parboiled Rice by Raman and NIR Spectroscopy

**DOI:** 10.3390/molecules30142938

**Published:** 2025-07-11

**Authors:** Ebrahim Taghinezhad, Antoni Szumny, Adam Figiel, Ehsan Sheidaee, Sylwester Mazurek, Meysam Latifi-Amoghin, Hossein Bagherpour, Natalia Pachura, Jose Blasco

**Affiliations:** 1 Biosystems Engineering Department, Faculty of Agriculture, Tarbiat Modares University, Tehran 14117-13116, Iran; 2 Department of Food Chemistry and Biocatalysis, Wroclaw University of Environmental and Life Sciences, 50-375 Wrocław, Poland; 3 Institute of Agricultural Engineering, Wroclaw University of Environmental and Life Sciences, 51-630 Wrocław, Poland; 4 Department of Chemistry, University of Wrocław, 50-383 Wrocław, Poland; 5 Department of Biosystems Engineering, College of Agriculture and Natural Resources, University of Mohaghegh Ardabili, Ardabil 56199-11367, Iran; 6 Department of Biosystems Engineering, Faculty of Agriculture, Bu-Ali Sina University, Hamedan 65178-33131, Iran; 7 Department of Environmental Hygiene and Animal Welfare, Wroclaw University of Environmental and Life Sciences, 51-630 Wrocław, Poland; 8 Centro de Agroingeniería, Instituto Valenciano de Investigaciones Agrarias, 46113 Valencia, Spain

**Keywords:** parboiled rice, Raman spectroscopy, starch gelatinization, head rice yield, aroma components

## Abstract

Vibrational spectroscopy, including Raman and near-infrared techniques, enables the non-destructive evaluation of starch gelatinization, head rice yield, and aroma-active volatile compounds in parboiled rice subjected to varying soaking and drying conditions. Raman and NIR spectra were collected for rice samples processed under different conditions and integrated with reference analyses to develop and validate partial least squares regression and artificial neural network models. The optimized PLSR model demonstrated strong predictive performance, with R^2^ values of 0.9406 and 0.9365 for SG and HRY, respectively, and residual predictive deviations of 3.98 and 3.75 using Raman effective wavelengths. ANN models reached R^2^ values of 0.97 for both SG and HRY, with RPDs exceeding 4.2 using NIR effective wavelengths. In the aroma compound analysis, *p*-Cymene exhibited the highest predictive accuracy, with R^2^ values of 0.9916 for calibration, and 0.9814 for cross-validation. Other volatiles, such as 1-Octen-3-ol, nonanal, benzaldehyde, and limonene, demonstrated high predictive reliability (R^2^ ≥ 0.93; RPD > 3.0). Conversely, farnesene, menthol, and menthone showed poor predictability (R^2^ < 0.15; RPD < 0.4). Principal component analysis revealed that the first principal component explained 90% of the total variance in the Raman dataset and 71% in the NIR dataset. Hotelling’s T^2^ analysis identifies influential outliers and enhances model robustness. Optimal processing conditions for achieving maximum HRY and SG values were determined at 65 °C soaking for 180 min, followed by drying at 70 °C. This study underscores the potential of integrating vibrational spectroscopy with machine learning techniques and targeted wavelength selection for the high-throughput, accurate, and scalable quality evaluation of parboiled rice.

## 1. Introduction

**Figure 1:**
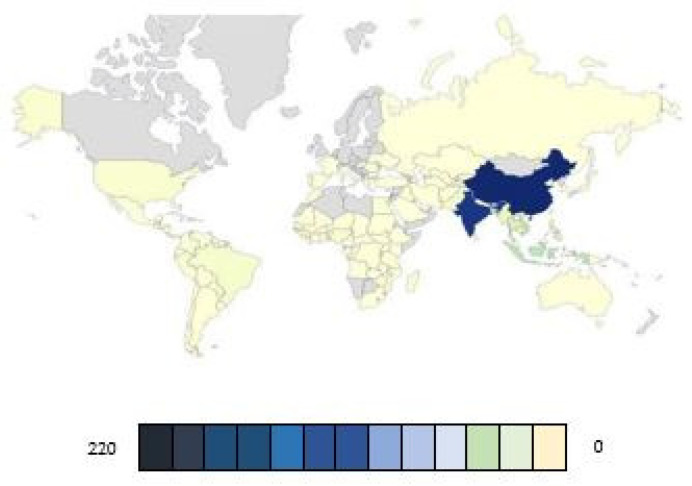
World rice production in kilotons.

Rice, a species of *Oryza sativa*, is part of the *Oryza* genus and the Poaceae family [1]. Rice plants belong to the grass species category and are extremely significant because of their edible grains [2]. This plant requires warm and humid climatic conditions for optimal growth and is commonly cultivated in paddy fields [3]. Rice features long, narrow leaves, hollow stems, and clusters that produce the rice grains [4]. Rice, as one of the most important food sources for humans, has contributed significantly to energy provision, with consumption exceeding 520 million tons in 2023/2024 [5]. Asian countries are the largest rice producers in the world. Among them, China ranks first, with a production of 208,495 kilotons. Following China, India holds the second position with a production of 196,246 kilotons, whereas Bangladesh ranks third with 57,189 kilotons. These three countries are pivotal in meeting global demand for rice (Figure 1). This food is rich in carbohydrates and supplies the energy required for daily activities [6]. One of the key benefits of rice is its gluten-free nature, making it a suitable choice for individuals with celiac disease [7,8]. Additionally, different types of rice offer various nutritional benefits; white rice, owing to its simpler structure, is easier to digest [9], while brown rice is rich in fiber, antioxidants, and nutrients, which can help reduce cholesterol and improve heart health [10]. Moreover, rice contains an appropriate amount of sodium, which contributes to blood pressure control [11]. This food also contains vitamin B, magnesium, and selenium, all of which are essential for maintaining overall health [12].

The chemical composition of rice includes proteins, lipids, free sugars, fatty acids, phytic acid, vitamin E, *γ*-oryzanol, and *γ*-aminobutyric acid (GABA) [13]. Germination significantly enhances the chemical composition and functional properties of brown rice, resulting in a notable increase in vitamin E, *γ*-oryzanol, and *γ*-aminobutyric acid, all of which contribute significantly to health promotion and disease prevention [13]. The components of the rice grain include the bran, bran layer, germ, endosperm, and aleurone layers [14,15]. The mean dimensions of the rice grain (*Oryza sativa* L.), including length, width, and thickness, are approximately 8.45 mm, 2.36 mm, and 1.86 mm, respectively [16]. In the evaluation of physical properties, processing of rice grains resulted in a 51% increase in the bulk density. The porosity of milled rice reached 26%, and both the static and dynamic friction coefficients were reduced [17]. A constant force was applied to different rice varieties, with the highest friction observed on wooden surfaces [17]. In another study, storage affected various properties of three common rice varieties, resulting in increased grain swelling, water absorption, bulk density, color intensity, and dough expansion, while reducing rice flour particle size and cooked grain stickiness; these effects were more pronounced at higher storage temperatures [18]. Parboiling extends the shelf life and enhances the nutritional value of rice, contributing significantly to improving its overall quality [19]. Several studies have investigated the parboiling process of rice, which results in reduced grain breakage [20] and enhanced nutritional value through changes in ash, lipid, fiber, protein, phytic acid, and antioxidant contents [21]. Near-infrared spectroscopy has been used to estimate the hardness and toughness of rice [22], and the effects of the steaming process on the crystallinity, texture, and mechanical properties of rice have been examined [23]. Additionally, the impact of the hydration stage during the parboiling process at 55 °C and 65 °C on starch gelatinization in paddy rice has been studied [24], along with investigations into mass transfer, heat transfer, and gelatinization during parboiling [25].

The aim of the present study was to evaluate the percentage of rice gelatinization, assess healthy rice yield, and identify volatile components following the parboiling process using Raman and NIR spectroscopy. Principal component analysis was utilized for dimensionality reduction, and PLSR models were developed to select the optimal parboiling method for rice. Additionally, the concept of the effective wavelength was applied to enhance the selection of key spectral variables contributing to the prediction accuracy of the models.

## 2. Results

### 2.1. SG and HRY Values

The starch gelatinization of the rice samples was examined after soaking at three different temperatures (60 °C, 65 °C, and 70 °C) and, subsequently, drying under ambient conditions without steaming. The results showed that SG values increased progressively from 8.78% to 60.54% as the soaking time and temperature increased. Notably, the highest SG value was observed at a soaking temperature of 70 °C and soaking duration of 120 min. These findings align with previous research indicating that rice SG tends to increase with the intensity of the parboiling treatment [26].

**Figure 2:**
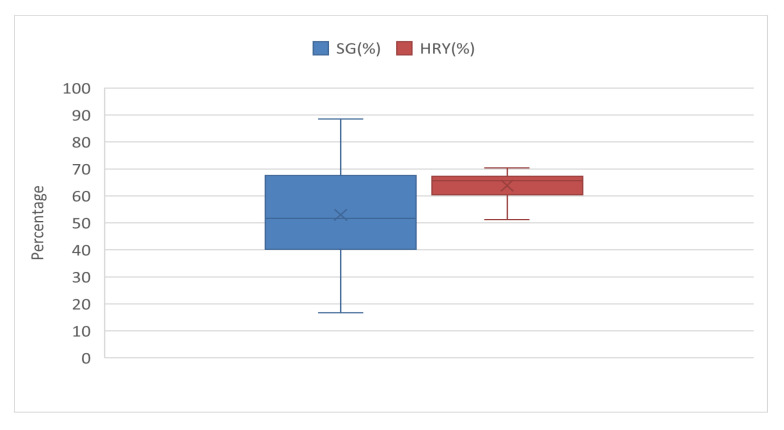
Percentage of HRY and SG in parboiled rice.

The increase in SG can be attributed to water absorption by starch granules during the soaking process. This absorption causes the starch granules to swell irreversibly and reduces their molecular weight, ultimately enhancing the SG value. Furthermore, the impact of the ICM drying method was investigated at three different temperatures (50, 60, and 70 °C) and drying durations. The samples used in this drying process were soaked under three different conditions: 60 °C for 240 min, 65 °C for 180 min, and 70 °C for 120 min, followed by steaming for 10 min. The results demonstrated that increasing the soaking temperature, drying temperature, and drying time led to an increase in the SG values from 40.83% to 88.51%. The SG value is an indicator of the parboiling process intensity (Figure 2) [27,28]. This relationship highlights that SG values increase in direct proportion to the intensity of the heat treatment during parboiling [29,30]. Similar findings have been reported by other researchers, who observed starch gelatinization during the drying process [31].

The relationship between the HRY, soaking temperature and time was analyzed. As soaking time increased from 15 to 240 min at 60 °C, 15 to 180 min at 65 °C, and 15 to 120 min at 70 °C, the HRY values improved from 45.03% to 55.20%. The highest HRY (55.20%) was observed at a soaking time of 180 min and temperature of 65 °C. This improvement in HRY can be attributed to the optimal moisture content of the rice, which facilitates greater starch gelatinization. Additionally, an increase in soaking temperature leads to more thorough starch gelatinization, thereby strengthening the resistance of the rice hull during the process, ultimately increasing the HRY [26]. However, the HRY decreased when the soaking temperature was increased from 65 °C to 70 °C. This is because exceeding the gelatinization temperature causes rice grains inside the hull to cook, which may lead to grain breakage during processing, thereby lowering the HRY. An increase in the HRY after parboiling was also linked to the attachment of the rice hull to the grain. During soaking, the hull opens, and gelatinization of rice starch during steaming makes the grain structure more rigid, which enhances HRY [32].

The combined effect of the soaking temperature and hybrid ICM dryer temperature on HRY was also examined for various drying times. With an increase in drying time from 10 to 120 min and drying temperature from 50 °C to 70 °C, HRY increased from 53.7% to 70.35%. The highest HRY (70.35%) was observed at a soaking temperature of 65 °C, drying temperature of 70 °C, and drying time of 50 min (Figure 2). These results are consistent with those of previous studies [33,34], which suggested that increasing the drying time and temperature enhances the SG and subsequently improves the HRY. However, excessively high soaking temperatures may cause water absorption by the rice grains inside the hull, resulting in overcooking and grain breakage, which decreases HRY. Furthermore, other studies have reported that combining different drying methods, such as microwave and infrared drying with fluidized bed drying, can enhance the HRY by 5 to 40% compared to using single drying methods [35].

### 2.2. Aroma Chemicals

The SPME-GC/MS analysis identified several key volatile compounds that contributed to the aroma profiles of the samples. The detected compositions included a diverse range of aldehydes, alcohols, ketones, and terpenes, all of which are known for their significant roles in flavor and fragrance characteristics (Table 1) and the aroma compositions in rice parboiling with active ingredient value (Table 2). Among the prominent aroma-active compounds, pentanal is recognized for its sharp, pungent odor with green and nutty notes [36], whereas benzaldehyde provides a distinct almond-like aroma often associated with nuts and seeds [37]. Sabinene, a terpene, exhibited a spicy, woody scent common in essential oils [38], whereas 1-Octen-3-ol delivered an earthy, mushroom-like fragrance [39]. Another notable terpene, myrcene, contributes to a strong herbal and citrus aroma frequently found in hops and bay leaves [40].

Hexyl acetate added a fruity and sweet aroma reminiscent of apples and pears [41], while *p*-cymene offered mild, pleasant citrus and herbal undertones. The widely recognized limonene provided a fresh, citrus aroma typically found in citrus fruit peels [42], and acetophenone enhanced the profile with a sweet, floral scent resembling jasmine or honeysuckle [43]. In addition, nonanal contributed a waxy, citrus-like scent that reinforced fresh and clean notes. Compounds such as menthone and menthol delivered minty and cooling fragrances, characteristic of mint oils [44], while Pulegone added strong minty notes, similar to peppermint oils [45].

Further enhancing the profile, decanal emits a powerful citrusy aroma akin to orange peels [46], and farnesene plays a vital role in adding floral scents with green, woody notes [47]. Dodecane provided a mild, waxy, and clean scent that completed the aromatic spectrum [48]. These identified aroma compounds played a significant role in defining the overall sensory profiles of the analyzed samples. Their presence and concentration are essential factors in determining the flavor quality, particularly in food and fragrance applications.

### 2.3. Raman Spectroscopy

**Figure 3:**
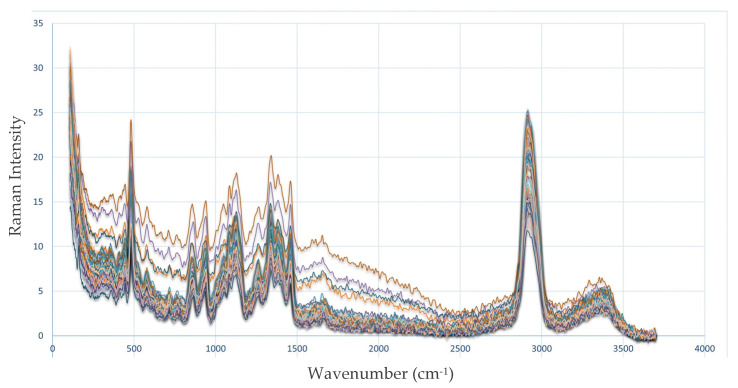
Raman spectra of parboiled rice samples. The spectra show parboiling positions at high repetitions.

Figure 3 presents the vibrational spectra obtained from the parboiled rice samples measured using Raman spectroscopy. Prior to analysis, outlier data points were identified and removed to ensure data accuracy. The Raman methods utilized a wavenumber range of 100 to 3700 cm^−1^, providing a comprehensive spectral profile for the examined samples.

**Figure 4:**
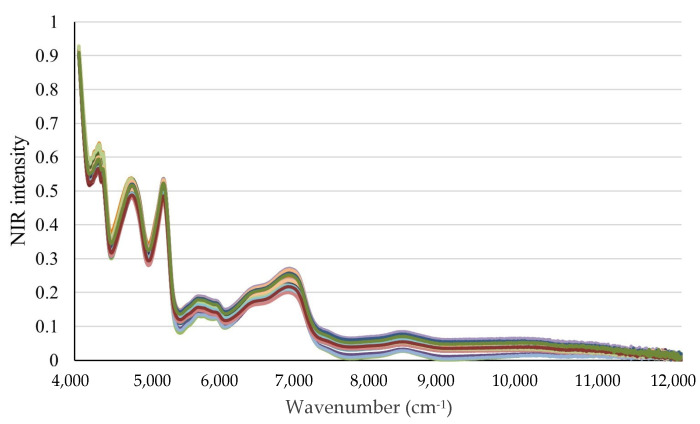
Reflectance of parboiled rice samples by NIR spectroscopy. The spectra show parboiling positions at high repetitions.

Figure 4 shows the reflectance spectra acquired from the parboiled rice samples measured within the NIR region using appropriate spectroscopic techniques. Prior to the spectral analysis, outliers were systematically identified and excluded to enhance data reliability. The NIR measurements covered a spectral range of 4000 to 12,000 cm^−1^, providing information about molecular vibrations through the observation of overtones and combination bands.

### 2.4. PCA

**Figure 5:**
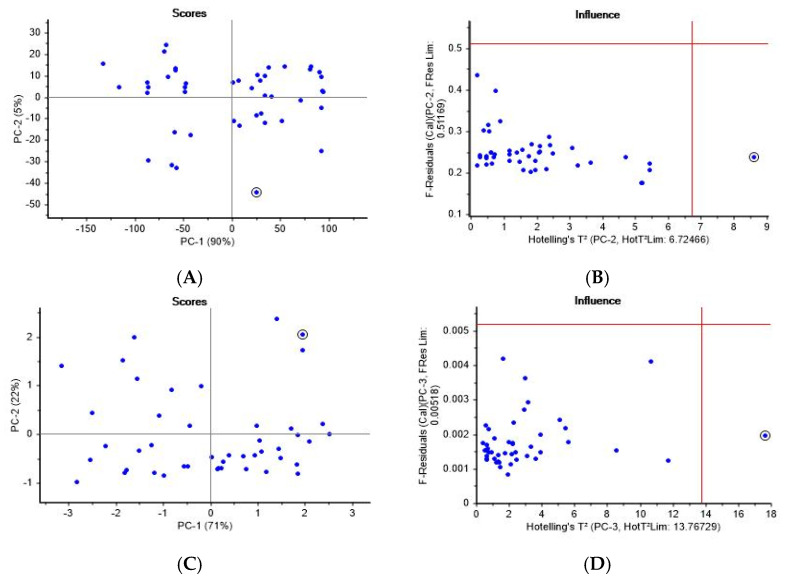
Principal components (PCs) from Raman and NIR data for parboiled rice: (**A**,**B**) Raman; (**C**,**D**) NIR.

In this study, the effectiveness of the principal component analysis (PCA) model was rigorously evaluated using Raman spectral data to extract meaningful patterns and reduce the dimensionality of the dataset. The application of PCA allowed for the identification of key spectral variations while minimizing redundant information, ensuring an optimal balance between data simplification and the retention of essential characteristics. Figure 5 presents the PCA results from the Raman and NIR spectral data for parboiled rice. Panels A and C depict the score plots, while Panels B and D show the corresponding influence plots, highlighting the application of Hotelling’s T^2^ statistic to identify outliers and assess the model fit. In the Raman data (Figure 5A), the score plot shows that the first principal component (PC1) accounted for 90% of the total variance (95%), capturing the most significant variation among the samples. For the NIR data, the score plot (Figure 5C) indicated that PC1 explained 71% of the total variance (93%), demonstrating a more distributed structure compared to the Raman data. Hotelling’s T^2^ statistic is a multivariate measure used to assess the distance of each sample from the centroid of the dataset in the reduced principal component space. In the influence plots (Figure 5B,D), Hotelling’s T^2^ was plotted on the x-axis, while the F-residuals were plotted on the y-axis. The red vertical lines in the plots correspond to critical Hotelling’s T^2^ thresholds (determined by Hotelling’s T^2^ limit), which highlight data points that are far from the centroid and could potentially be outliers. In both the Raman (Figure 5B) and NIR (Figure 5D) data, several samples exceeded Hotelling’s T^2^ threshold, indicating that these samples deviated significantly from the overall data distribution. In Figure 5B, for the Raman data, the threshold was set at 6.72466, and a few samples exceeded this limit, suggesting that these data points had a higher degree of influence on the PCA model than the others. These outliers may represent distinct characteristics of the samples, which warrant further investigation or may be due to experimental errors. For the NIR data in Figure 5D, the threshold was set at 13.76729, and some samples were identified as influential outliers based on their high Hotelling’s T^2^ values. These samples have a greater impact on the overall model structure and must be carefully reviewed, as their inclusion could distort subsequent analyses, such as classification or regression. The application of Hotelling’s T^2^ in the influence plots plays a critical role in identifying outliers, ensuring that the PCA model reflects the true variability of the data. By setting appropriate thresholds, the influence plots help determine which samples exert disproportionate influence on the principal components and allow for the adjustments or removal of problematic data points to enhance the robustness of the model.

### 2.5. Regression Model Based on PLSR

**Figure 6:**
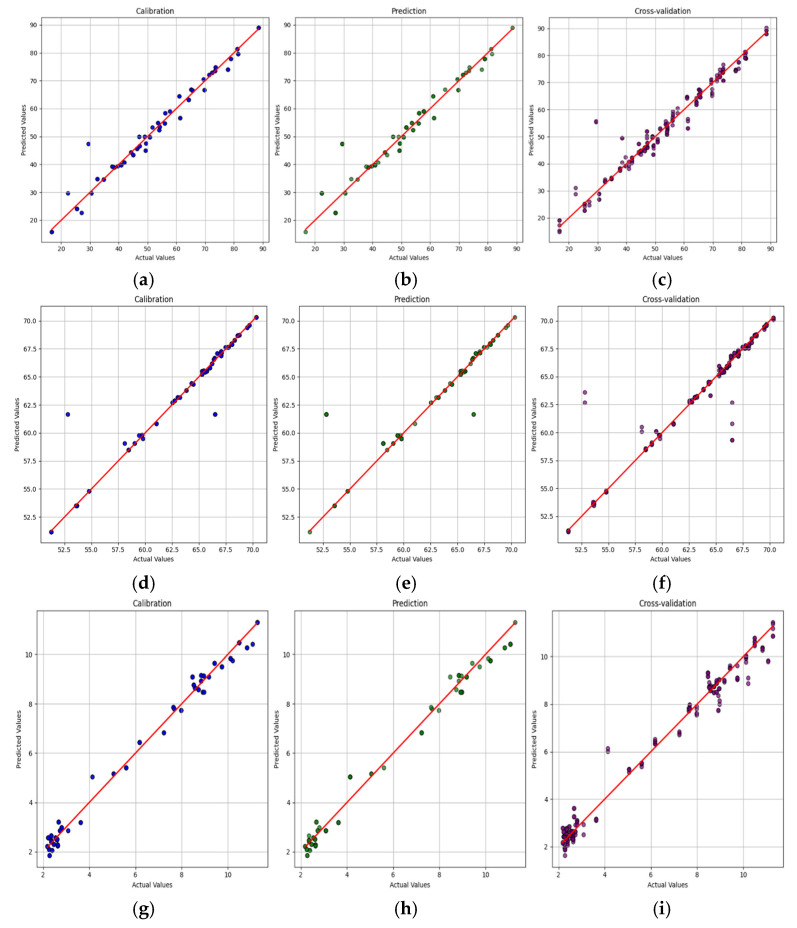
Prediction plot for Raman spectra: (**a**–**c**) HRY; (**d**–**f**) SG; (**g**–**i**) *p*-Cymene.

The predictive performance of the spectral models for detecting the aroma components was evaluated using calibration, prediction, and cross-validation metrics. The dataset was split into 70% for calibration and 30% for prediction, and five-fold cross-validation was applied to ensure model robustness. Among the tested aroma compounds, *p*-cymene showed the highest predictive accuracy, making it the most reliable compound for aroma detection. It achieved an R^2^_C_ of 0.9916, R^2^_P_ of 0.9859, and R^2^_CV_ of 0.9814, indicating excellent consistency across different data subsets (Figure 6g–i). Furthermore, it exhibited the lowest root mean square error of calibration (RMSE_C_ = 0.0295) and prediction (RMSE_P_ = 0.0392) as well as the highest residual predictive deviation (RPD = 10.93), confirming its strong quantitative predictive capability. These results suggest that *p*-cymene is highly suitable for accurately detecting aroma components, surpassing the other tested volatiles in terms of predictive performance (Table 3).

In contrast, HRY and SG showed comparatively lower performances. HRY had an R^2^_C_ of 0.9412, but its R^2^P dropped to 0.8137, suggesting reduced accuracy when applied to unseen data (Figure 6a–c). The five-fold cross-validation R^2^ was 0.9119, indicating moderate model reliability. Higher RMSE_P_ (2.1285) and RMSE_CV_ (1.4252) values resulted in a lower RPD of 4.12, making it suitable for rough screening, but less reliable for precise aroma quantification. Similarly, SG performed better than HRY, with an R^2^_P_ of 0.9190 and a higher RPD of 6.56, indicating a more reliable but still improvable predictive model (Figure 6d–f).

Despite these variations, all the tested aroma compounds exhibited strong cross-validation performance, with R^2^_CV_ values exceeding 90% and RMSE_CV_ values under 2, confirming the overall robustness of the spectral models. For example, benzaldehyde, limonene, and pulegone displayed R^2^_CV_ values above 0.95, coupled with low RMSE_CV_ values (≤0.13), suggesting high reliability for aroma component detection. Similarly, hexyl acetate, menthol, and nonanal showed consistent performance, with R^2^_CV_ values above 0.92 and RMSE_CV_ values, indicating strong predictive capability with minimal errors. These results suggest that although *p*-cymene remains the benchmark for aroma detection, several other compounds also exhibit excellent predictive performance and can be effectively utilized for aroma profiling in practical applications.

The predictive performance of PLS regression models developed using NIR spectra (4000–12,000 cm^−1^) for parboiled rice volatiles was evaluated through calibration, cross-validation, and residual predictive deviation (RPD) metrics. Among the analytes, 1-octen-3-ol, nonanal, and pentanal demonstrated strong quantitative predictive capabilities, with R^2^ values exceeding 0.93 for both calibration and cross-validation phases, and RPD values above 2.85, confirming their suitability for reliable aroma quantification. *p*-cymene also exhibited high model robustness (R^2^_CV_ = 0.8499, RPD = 2.66), indicating dependable performance across validation sets. In contrast, compounds such as farnesene, menthol, and menthone yielded very low R^2^ and RPD values (R^2^_CV_ < 0.2; RPD < 0.2), reflecting poor model reliability and limited predictive potential. Dodecane and pulegone showed moderate calibration R^2^ values (0.63 and 0.44, respectively) but had lower RPDs (≤2.15), rendering them less effective for precise predictions. The performance of bulk quality traits, such as HRY and SG, also varied; HRY showed moderate predictability (R^2^_CV_ = 0.7773, RPD = 2.43), whereas SG achieved higher accuracy (R^2^_CV_ = 0.9696, RPD = 3.03), supporting its application in routine quality control (Table 4). Overall, these results underscore the utility of NIR-based models for the nondestructive evaluation of aroma-related and physicochemical traits in parboiled rice, with several volatiles demonstrating high reliability and practical applicability.

### 2.6. Effective Wavelength

#### 2.6.1. Finding Effective Wavelengths from Raman and NIR Spectroscopy Data

The effective wavelengths were modeled using PLSR and ANN methods, which were applied to the wavelengths extracted through the learning automata (LA) algorithm [49]. To further enhance the precision of wavelength selection, a support vector machine-based learning automata (SVM-LA) algorithm was employed, which successfully identified ten effective wavelengths. The selected wavelengths demonstrated strong predictive capabilities for important quality parameters, including SG, HRY, and various aroma components. The integration of SVM with LA enabled a more refined and accurate wavelength-selection process, thereby improving the performance and robustness of the predictive models (Table 5).

#### 2.6.2. Effective Wavelength Modeling for Prediction

##### PLSR

The performance of PLSR models based on Raman and NIR spectroscopy, using effective wavelengths (EWs) selected by a support vector machine–learning automata (SVM-LA) algorithm, was rigorously evaluated for the prediction of HRY, SG, and key aroma compounds in parboiled rice. The incorporation of EWs significantly enhanced model accuracy by reducing spectral redundancy and focusing on the most informative spectral features. Raman-based models demonstrated strong calibration and validation results for traits such as SG and HRY, with SG achieving R^2^ values of 0.9406 and 0.9365 and RPDs of 3.98 and 3.75, respectively, indicating excellent predictive performance when using optimized EWs. Similarly, several volatile compounds such as benzaldehyde and nonanal, showed high predictive reliability (R^2^ ≥ 0.86; RPD ≥ 3.46), confirming that the selection of EWs enhances their quantification using Raman spectra. In contrast, the prediction of compounds farnesene and menthone remained poor (R^2^ < 0.1; RPD < 0.3), suggesting that these volatiles may lack sufficient Raman-active signals or that their spectral contributions fall outside the selected EWs. NIR-based PLSR models also benefited from EW optimization, showing improved performance for volatiles such as 1-Octen-3-ol, nonanal, and pentanal, all of which achieved R^2^ values above 0.93 and RPDs greater than 2.70 in both the calibration and validation phases. However, a low predictive capacity was still observed for menthol and pulegone, with R^2^ values below 0.05, and RPDs under 1.0, highlighting their limited suitability despite wavelength selection (Table 6).

Strong predictive capabilities were observed for volatiles such as 1-octen-3-ol, nonanal, and pentanal, each achieving calibration and validation R^2^ values exceeding 0.93, and RPDs above 2.70, confirming their quantifiability using selected NIR data. Similarly, benzaldehyde and limonene demonstrated robust model stability with validation R^2^ values of 0.9404 and 0.9085 and corresponding RPDs of 2.75 and 2.66, respectively (Table 6). SG showed excellent model performance (R^2^ = 0.9743; RPD = 2.85), while HRY exhibited moderate predictability (R^2^ = 0.787; RPD = 2.30), underscoring the relevance of NIR-EW models in routine quality control. In contrast, poor predictive outcomes were observed for compounds such as farnesene, menthol, and menthone, with validation R^2^ values below 0.15 and RPDs below 0.4, suggesting minimal spectral contribution in the selected NIR range.

##### ANN

The predictive capability of artificial neural network models built on Raman spectral data (100–3700 cm^−1^), incorporating effective wavelengths selected via the SVM-LA algorithm, was comprehensively assessed for the estimation of HRY, SG, and a suite of aroma-active volatiles in parboiled rice. The integration of EWs into the ANN framework notably enhances model precision by directing learning toward spectrally informative regions while reducing noise from redundant bands. Physicochemical parameters such as HRY and SG displayed high predictive reliability, with test R^2^ values of 0.94 and 0.89 and RPDs of 3.66 and 3.91, respectively, demonstrating the model’s robustness across independent sets. Among the aroma volatiles, benzaldehyde, 1-octen-3-ol, and hexyl acetate yielded consistent and high predictive performance, with R^2^ values above 0.72 and RPDs exceeding 3.50 on test data. Volatiles such as *p*-cymene, pentanal, and limonene also showed strong ANN predictability, with test R^2^ ≥ 0.84 and RPDs ranging from 3.27 to 3.63, indicating that Raman–EWs coupling is well-suited for volatile quantification. However, compounds such as farnesene, menthol, and menthone performed poorly, with test R^2^ values below 0.10 and RPDs under 0.30, reflecting insufficient Raman signal contributions or the inability of ANN to generalize from limited spectral features. These findings reinforce the potential of combining Raman spectroscopy with EW-ANN approaches for the non-destructive, rapid assessment of rice quality and aroma, while also emphasizing compound-specific limitations that warrant further exploration (Table 7).

The predictive performance of artificial neural network models was constructed using NIR spectral data (4000–12,000 cm^−1^), and key quality parameters such as HRY and SG demonstrated excellent predictive accuracy, with test R^2^ values of 0.97 and 0.97 and RPDs of 4.21 and 4.41, respectively, showcasing the strength of the NIR-ANN approach for physical trait estimation (Table 7). Similarly, aromatic compounds such as 1-octen-3-ol, benzaldehyde, limonene, and pentanal were predicted with high reliability, all achieving test R^2^ values above 0.96 and RPDs greater than 4.20, indicating a strong correlation between NIR spectral features and their chemical signatures. Compounds, including hexyl acetate, *p*-cymene, and sabinene also yielded robust model outputs (R^2^ ≥ 0.94; RPD ≥ 4.16), further confirming the suitability of NIR-based ANN models for complex volatile profiling. Conversely, volatiles such as farnesene, menthol, and menthone exhibited low predictability (test R^2^ < 0.15; RPD < 1.0), suggesting limited NIR absorbance in the chosen spectral range or insufficient spectral variance to support accurate modeling. Collectively, these findings underscore the potential of combining NIR spectroscopy with machine learning techniques, such as ANN and SVM-LA-driven EWs selection, for the efficient, high-throughput evaluation of rice quality and aroma, as well as identifying specific volatiles that may require alternative sensing methods.

In conclusion, the use of support vector machine–learning automata (SVM-LA) for selecting EWs significantly improved the prediction accuracy of quality parameters and volatile compounds in parboiled rice when analyzed using Raman and NIR spectroscopy. By identifying the most informative wavelengths, the SVM-LA approach reduces spectral redundancy and optimizes the input variables for the PLSR model. The selected EWs enabled high predictive performance for SG, HRY, and key aroma compounds like 1-octen-3-ol, benzaldehyde, and nonanal, with several models achieving R^2^ values above 0.93 and RPDs exceeding 3.0. These findings affirm that combining SVM-LA with spectroscopy enhances model robustness and efficiency, making it a valuable tool for nondestructive quality control in rice processing. However, the models struggled to predict compounds such as farnesene, menthol, and menthone, indicating either their limited Raman or NIR responsiveness, or the need for alternative wavelength regions not captured by the selected EWs.

## 3. Discussion

SG and HRY analyses demonstrated that soaking temperature, drying conditions, and processing duration significantly influenced rice quality, with SG values increasing with soaking temperature and durations. However, the HRY declined beyond 65 °C, indicating that excessive heat exposure weakened the grain structure, leading to breakage. The study also explored hybrid drying techniques, revealing that increased drying temperatures and durations positively influenced HRY; however, extreme conditions caused quality deterioration. Additionally, aroma profiling identified key volatile compounds, such as benzaldehyde, limonene, and *p*-cymene. These findings highlight the importance of balancing thermal treatment, drying efficiency, grain stability, and aroma retention to enhance rice yield, milling recovery, and overall consumer appeal.

## 4. Materials and Methods

### 4.1. Sample Preparation

For this study, a sample of paddy rice (rice before husking or milling) weighing 10 kg was procured from the Baibor company (Sevilla, Spain). The selected variety, known as PUNTAL, is characterized by its distinct physical dimensions, with an average grain length of 6 mm and an average width of 3 mm. Prior to the experimental process, the initial moisture content of the paddy was measured to be 12% on a wet basis (w.b.). A total of 43 rice samples were analyzed, and each sample was subjected to triplicate spectrometric measurements to ensure data reliability and reproducibility.

To ensure the stability of the moisture content throughout the research process, the paddy samples were stored under controlled environmental conditions. This was achieved by placing the samples in a climatic chamber (Memmert TCH 750, Schwabach, Germany) set to maintain a stable temperature of 24 ± 1 °C and relative humidity of 60 ± 1%, as recommended by the International Rice Research Institute (IRRI, 2022).

To accurately determine the moisture content of the samples, a standard oven drying method was employed. Specifically, three separate 10 g samples of the paddy were weighed and then placed in a drying oven (Model D06836, Memmert Company, Schwabach, Germany). The samples were heated to 130 °C for 24 h. This method ensured the precise and consistent measurements of the moisture content (Equation (1)), which is a crucial parameter in the subsequent stages of research. The masses of the samples were accurately measured using a high-precision digital scale (AND GF-600, Osaka, Japan), which provided a measurement accuracy of 0.001 g.

(1) $$M.C.=\frac{m_{1}-m_{2}}{m_{1}}\times 100$$

The moisture content was calculated using the Equation (1), where m_1_ represents the initial sample mass and m_2_ denotes the final mass after drying.

### 4.2. Parboiling Process

Parboiling involves two distinct methods. In Method 1 (Table 8), 200 g of paddy samples was soaked in 1000 mL of distilled water using a temperature-controlled water bath (GT0610, Shenzhen, China) at three temperatures: 60 °C, 65 °C, and 70 °C, for 240, 180, and 120 min, respectively, until the moisture content reached approximately 35% (wet basis). At each temperature, samples were collected at five time intervals, coded for clarity, and dried in the shade under controlled ambient conditions (27 ± 1 °C, 20 ± 5% RH) until a final moisture content of ~15% (w.b.) was achieved. In Method 2 (see Table 8), the same soaking conditions were applied, followed by a 2 h rest period and steaming at 97 °C for 10 min using a Perfect Cook pressure cooker (SW500, Eldom, Dalian, China).

The steamed samples were then dried in a hybrid ICM dryer developed in the post-harvest technology laboratory at three drying temperatures (50, 60, and 70 °C), with an inlet airspeed of 1 m/s, until they reached ~15% moisture content. After drying, all samples were transported to the Heat Transfer Laboratory at the Wrocław University of Environmental and Life Sciences for husking, milling, and measurement of specific gravity (SG) and head rice yield (HRY), ensuring consistency in the evaluation process. For additional analysis, the rice samples were sent to the Biosystem Laboratory at the Valencian Institute for Agricultural Research (IVIA), Spain, where Raman spectroscopy was conducted. All experiments were performed in triplicates to ensure data reliability. The hybrid ICM dryer used in this study integrated two temperature sensors to regulate three heat sources: convection (via a controlled inlet air temperature), infrared, and microwave heating. These sensors automatically activated or deactivated the heat sources based on the internal chamber conditions. During each drying test, 200 g of paddy was spread in a 3–5 mm layer on the trays inside the dryer. The sample weight was continuously monitored using a precision digital scale (AND GF-600, Japan; ±0.001 g accuracy) with data recorded by a computer. The environmental conditions were maintained (20 ± 5 °C and 32 ± 5% RH). As detailed in Table 8, 40 g of sample was collected at five specific time intervals during each drying run for further laboratory analysis.

### 4.3. Starch Gelatinization of Rice

The conversion process was conducted on all dried rice samples prior to the commencement of the experiments. This multistep conversion procedure consists of two distinct stages: husking and milling. The husking stage was performed using a paddy husker machine (NA.JLG-2018, ACME AGRO-TECH CO., Qiaokou, China), followed by the milling stage, which was performed using a milling machine (LTJM-160, ACME AGRO-TECH CO., China). To assess the gelatinization of rice starch, the method outlined by Taghinezhad et al., 2016 was used [26]. Specifically, the degree of gelatinization was determined using a differential scanning calorimeter (DSC 821e, Mettler Toledo, Greifensee, Switzerland) within a temperature range of 25–100 °C at a heating rate of 5 °C/min. The percentage of gelatinized starch was calculated using the equation provided in Equation (2) [27].

(2) $$SG\;\left(\%\right)=\left[1-\frac{∆H}{{∆H}^{*}}\right]\times 100$$

In this context, ΔH and ΔH∗ represent the enthalpy changes associated with the gelatinization of rice starch. Specifically, ΔH refers to the enthalpy change that occurs during the gelatinization of rice starch, reflecting the energy absorbed as starch undergoes structural transformation. On the other hand, ΔH∗ denotes the enthalpy change in the raw, non-gelatinized rice starch, which serves as the baseline enthalpy before any thermal or structural modifications take place during the heating process.

### 4.4. Head Rice Yield of Rice

To assess the head rice yield, paddy samples were weighed before milling. The milling operation includes two primary steps: husking and whitening. Following these steps, milled rice was transferred to a centrifugal separator (FQS-13X20, Sensewealth, Changde, China) for further classification.

During the separation process, broken rice grains were effectively separated from the head rice. In this context, head rice refers to grains that measure at least 75% of the length of a complete unbroken grain. The head rice yield was calculated by dividing the number of head rice grains by the total number of processed paddy samples. This method followed the procedure described by Bello et al., 2006, to ensure accuracy and consistency in determining HRY values [34].

### 4.5. Spectroscopic Instruments

Raman measurements were conducted using a Raman accessory equipped with a CaF_2_ beamsplitter and an indium–gallium–arsenide detector. The samples were illuminated by a 1.064 μm Nd:YVO_4_ laser, delivering 150 mW of power to the sample. The backscattered radiation was collected for analysis. Each interferogram was averaged over 256 scans and then processed using Happ–Genzel apodization, Mertz phase correction, and Fourier transformation, with a zero-filling factor of 2. This process produced spectra in the 100–3700 cm^−1^ range with a resolution of 8 cm^−1^. The analyzed samples were prepared as pellets by pressing 150 mg of homogenized pollen under a pressure of 5 T/cm^2^. During measurements, the pellets were rotated at a constant speed of 200 rpm to ensure uniform data collection.

The vibrational spectra of homogenized parboiled rice samples were acquired using an iS50 Fourier transform infrared (FTIR) spectrometer (Thermo Nicolet, Madison, WI, USA).

### 4.6. HS-SPME-Arrow Aroma Compound

VOCs extraction from rice samples was performed using the HS-SPME-Arrow technique with a 2 cm fiber (DVB/CAR/PDMS, Supelco, Bellefonte, PA, USA). One gram of homogenized powdered rice was weighed into a 20 mL headspace glass vial. Subsequently, 10 µg of 2-undecanone (Labiochem s.c., Wrocław, Poland) was added as the internal standard. The samples were preincubated at 60 °C for 10 min. Arrow fiber was exposed to the headspace for 30 min, and the analytes were thermally desorbed in the injector at 250 °C for 3 min. Analyses were performed on a GC-MS system (Shimadzu, Kyoto, Japan) equipped with a Zebron ZB-5 MSi column (30 m × 0.25 mm × 0.25 µm; Phenomenex, Torrance, CA, USA). The oven temperature program was as follows: initial temperature 50 °C, ramped at 4 °C·min^−1^ to 130 °C, then 10 °C·min^−1^ to 180 °C, and finally 20 °C·min^−1^ to 280 °C. Mass spectra were recorded in electron impact (EI) mode at 70 eV in the 40–400 m/z range, with a scan rate of 3 scans·s^−1^. Samples were injected in split mode (1:10), using helium as the carrier gas at a flow rate of 1.0 mL·min^−1^. Retention indices (RIs) were calculated using a homologous series of *n*-alkanes (C7–C24) to support compound identification. Spectral data were matched against entries in the NIST mass spectral library, and compound assignments were further confirmed through RI comparison. Quantitative data were derived from the integrated peak areas using Spectrus software (2024, https://www.acdlabs.com/products/spectrus-platform/, accessed on 8 July 2025) (ACD/Labs), ensuring the accurate profiling of the aroma compound composition.

### 4.7. Statistical Analysis

#### Partial Least Squares Regression (PLSR)

The Partial Least Squares Regression method, derived from the PLS technique, offers a structured multivariate framework to describe the relationship between two datasets: X and Y. In this framework, the X dataset includes instrumental data (spectra bond), whereas the Y dataset corresponds to the target responses requiring modeling (rice labels) [50]. PLSR is particularly effective in quantitatively modeling complex relationships between predictor variables (X) and response variables (Y). This advantage becomes apparent when the X data exhibit high correlation-, noise-, or model-associated errors. These features make PLSR more robust and practical for real-world data analysis than the multiple linear regression (MLR) method, which assumes error-free X variables [51]. To determine the optimal number of latent variables (LVs) in the PLSR model, a validation process based on the minimum root mean squared error of cross-validation (RMSECV) was employed. The RMSECV was calculated for models with different LV counts, and the model with the lowest RMSECV value was selected as the optimal model, ensuring the best predictive performance with minimal overfitting. The appropriate selection of LVs is crucial for model accuracy. Using too few LVs may result in underfitting where essential data patterns are overlooked. Conversely, an excessive number of LVs can introduce noise, thereby reducing the ability of the model to generalize effectively. The performance of the PLSR model was assessed using two key metrics: coefficient of determination (R^2^) and root mean squared error. While R^2^ measures the accuracy of the linear regression fit, the RMSE is considered a reliable indicator of model quality, particularly in chemometric analysis. To enhance model performance, a five-fold cross-validation procedure was conducted on the calibration set samples. The R^2^ and RMSE values were calculated separately for calibration, prediction, and cross-validation using Equations (3) and (4), respectively.

(3) $$R^{2}=1-\left(\frac{\sum_{k=1}^{M}\;(Y_{e}-Y_{p})}{\sum_{k=1}^{M}\;\left(Y_{e}-Y_{a}\right)}\right)$$

(4) $$RMSE={\left(\frac{\sum_{k=1}^{M}\;(Y_{e}-Y_{p})}{M}\right)}^{1/2}$$

In this context, M denotes the total number of spectra involved in the calibration, prediction, and cross-validation processes. The variable Ye signifies the experimentally determined rice content, whereas Yp represents the rice content predicted by the PLSR model. Ya refers to the mean value of all experimental content measurements.

## 5. Conclusions

In conclusion, this study emphasizes the effectiveness of integrating advanced spectroscopic techniques and chemometric modeling to assess rice quality, with a particular focus on starch gelatinization and head rice yield. The results confirm that soaking temperature and duration are essential factors, with the highest SG achieved at 70 °C for 120 min, and the optimal HRY observed at a soaking temperature of 65 °C followed by drying at 70 °C. Raman spectroscopy, combined with principal component analysis (PCA), proved to be a powerful approach for monitoring molecular changes during parboiling, successfully identifying key spectral markers associated with SG and HRY.

In addition, this study demonstrates the strong predictive capabilities of spectral models, particularly PLSR and ANN, in quantifying quality traits and aroma volatiles. Notably, the aroma compound *p*-cymene exhibited exceptional model performance, with R^2^ values exceeding 0.98 and RPD scores, highlighting its reliability as a marker for aroma profiling. Other volatiles such as 1-octen-3-ol, benzaldehyde, and nonanal, also showed high predictability, particularly when effective wavelengths were selected using the SVM-LA algorithm. However, volatiles, such as farnesene, menthol, and menthone demonstrated poor predictability, likely due to weak spectral responses.

These findings underscore the potential of coupling spectroscopy with machine learning and intelligent wavelength selection for non-destructive, high-throughput quality evaluation in rice. The robustness of the models affirms the value of this approach for enhancing both the sensory and nutritional attributes of rice through improved processing control and quality monitoring. Overall, the methodology presented herein offers a scalable solution for future rice quality assessments and optimization strategies.

## Figures and Tables

**Table 1:** List of chemical compounds of parboiled rice.

Alcohol Compounds	Value (µg/kg)	Aldehyde Compounds	Value (µg/kg)	Ketone Compounds	Value (µg/kg)
*1*-Pentanol	15.72	Hexanal	229.6	Heptan-2-one	9.13
*n*-Hexanol	17.75	Pentanal	15.21	6-Methyl-Hept-5-en-2-one	10.14
*n*-Heptanol	1.52	*n*-Heptanal	58.82	Oct-3-en-2-one	47.66
*1*-Octen-3-ol	65.41	Benzaldehyde	63.38	Acetophenone	8.11
Menthol	40.56	*n*-Octanal	6.08	Menthone	1.01
Ester Compounds	Value (µg/kg)	*n*-Nonanal	259.61	Terpene and Aromatic Hydrocarbon Compounds	Value (µg/kg)
Hexyl acetate	42.59	*n*-Decanal	84.82	Sabinene	5.58
Hydrocarbon Compounds	Value (µg/kg)	(2*E*)-Nonenal	140.96	Myrcene	17.75
Octane, 4-methyl-	1.52	Dec-(2*E*)-enal	180.51	*p*-Cymene Limonene	141.47 66.42
*n*-Undecane	511.61	Phenolic and Aromatic Compounds	Value (µg/kg)		
Dodecane	1210.3	Benzene, 1-(1*E*)-1-butenyl-4-methoxy-	9.13	Pulegone	2.03
*n*-Tetradecane	105.97	Sulfur Compounds	Value (µg/kg)	Verdoracine	3.55
		Disulfide, dimethyl	1.01	*(E,E)-α*-Farnesene	1.01
				Perhydrofarnesyl acetone	1.01

**Table 2:** List of aroma chemical compounds of parboiled rice.

Chemical Compound	Value (µg/kg)	Chemical Compound	Value (µg/kg)
Pentanal	15.21	Sabinene	5.58
Limonene	66.42	Nonanal	259.61
Dodecane	1210.32	Menthone	1.01
Benzaldehyde	63.38	Menthol	40.56
Acetophenone	8.11	Myrcene	17.75
1-Octen-3-ol	65.41	Pulegone	2.03
Hexyl acetate	42.59	Decanal	94.82
Farnesene	1.01	Cymene (para-)	141.47

**Table 3:** PLSR model parameters for predicting HRY, SG, and aroma components of Raman data in parboiled rice.

Raw Data	LV	Range (cm^−1^)	R^2^_C_	R^2^_p_	R^2^_CV_	RMSE_C_	RMSE_P_	RMSE_CV_	RPD
HRY	7	100–3700	0.9412	0.8137	0.9119	1.1641	2.1285	1.4252	4.12
SG	5	100–3700	0.9767	0.9190	0.9542	2.6628	4.7969	3.7395	6.56
1-Octen-3-ol	5	100–3700	0.9726	0.9551	0.9528	0.0751	0.1083	0.0986	6.04
Acetophenone	5	100–3700	0.9777	0.9781	0.9565	0.194	0.1120	0.1132	6.70
Benzaldehyde	5	100–3700	0.9746	0.9410	0.9525	0.0427	0.0654	0.0584	6.27
*p*-Cymene	5	100–3700	0.9916	0.9859	0.9814	0.0295	0.0392	0.0440	10.93
Decanal	5	100–3700	0.9579	0.9183	0.9201	0.1012	0.1597	0.1395	4.87
Dodecane	5	100–3700	0.9842	0.9524	0.9714	1.2943	2.1705	1.7455	7.98
Farnesene	5	100–3700	0.9792	0.9468	0.9454	0.267	0.2113	0.2108	6.94
Hexyl acetate	5	100–3700	0.9816	0.9301	0.9652	0.3711	0.7316	0.5114	7.38
Limonene	5	100–3700	0.9884	0.9586	0.9761	0.1561	0.3115	0.2243	9.30
Menthol	5	100–3700	0.9811	0.9026	0.9497	0.1186	0.1347	0.1304	7.28
Menthone	5	100–3700	0.9759	0.9299	0.9420	0.222	0.239	0.235	6.45
Myrcene	5	100–3700	0.9651	0.8745	0.9288	0.0352	0.0767	0.0504	5.35
Nonanal	5	100–3700	0.9832	0.9222	0.9695	0.2706	0.4793	0.4645	7.72
Pentanal	5	100–3700	0.9791	0.9452	0.9476	0.0294	0.0489	0.0467	6.92
Pulegone	5	100–3700	0.9848	0.9560	0.9601	0.0261	0.0456	0.0453	8.11
Sabinene	5	100–3700	0.9594	0.9227	0.9173	0.1803	0.112	0.1114	4.96

**Table 4:** PLSR model parameters for predicting HRY, SG, and aroma components of NIR data in parboiled rice.

Raw Data	LV	Range “cm^−1^”	R^2^_C_	RMSE_C_	RPD	R^2^_CV_	RMSE_CV_	RPD
HRY	3	4000–12,000	0.8066	2.1259	2.74	0.7773	2.3335	2.43
SG	3	4000–12,000	0.9763	2.5646	3.32	0.9696	2.9727	3.03
1-Octen-3-ol	3	4000–12,000	0.9629	0.0911	3.27	0.9558	0.1016	2.99
Acetophenone	3	4000–12,000	0.881	0.0239	2.99	0.8526	0.0272	2.66
Benzaldehyde	3	4000–12,000	0.9484	0.061	3.22	0.9362	0.0694	2.93
*p*-Cymene	3	4000–12,000	0.8729	1.1614	2.97	0.8499	1.2909	2.66
Decanal	3	4000–12,000	0.8903	0.1712	3.03	0.8683	0.1919	2.71
Dodecane	3	4000–12,000	0.6313	6.232	2.15	0.5613	6.9519	1.75
Farnesene	3	4000–12,000	0.0331	0.0466	0.11	NA	0.0516	0.12
Hexyl acetate	3	4000–12,000	0.8619	1.0219	2.93	0.82	1.1934	2.56
Limonene	3	4000–12,000	0.9165	0.4269	3.11	0.9015	0.4744	2.82
Menthol	3	4000–12,000	0.0527	0.1258	0.18	NA	0.1378	0.17
Menthone	3	4000–12,000	0.0474	0.0143	0.16	NA	0.0158	0.15
Myrcene	3	4000–12,000	0.8582	0.0746	2.92	0.8334	0.0827	2.60
Nonanal	3	4000–12,000	0.9339	0.5179	3.17	0.9139	0.6045	2.86
Pentanal	3	4000–12,000	0.9387	0.0511	3.19	0.9205	0.0595	2.88
Pulegone	3	4000–12,000	0.444	0.0159	1.51	0.3781	0.0172	1.18
Sabinene	3	4000–12,000	0.9035	0.0127	3.07	0.8897	0.0139	2.78

**Table 5:** Wavelengths extracted by the SVM-LA method from Raman and NIR data to predict SG, HRY, and aroma components.

Raw Data	Selected EWs (cm^−1^)—Raman (100–3700 cm^−1^)	NIR (4000–12,000 cm^−1^)
HRY	824.35, 3562.8, 2313.2, 3024.8, 3261, 2908.1, 2408.6, 3163.6, 2289, 3263.9	6915, 4239, 5199, 7674, 6865, 4744, 6773, 6912, 5200, 11,582
SG	2801.1, 2784.7, 2041.2, 1236.1, 3351.7, 1165.7, 3353.6, 3436.5, 3396, 2328.6	6999, 6961, 6948, 6923, 10,534, 6745, 4665, 4907, 6572, 4274
1-Octen-3-ol	3616.8, 2307.4, 2010.4, 1662.3, 3259.1, 1731.7, 2388.4, 2796.2, 2549.4, 3650.6	6747, 4389, 6797, 6932, 5379, 4433, 6875, 4446, 6924, 9819
Acetophenone	3522.3, 872.57, 2373.9, 3089.4, 3154.9, 3524.3, 651.75, 2777, 2508.9, 2337.3	6632, 4461, 5049, 4848, 6880, 4571, 4867, 4835, 6819, 6974
Benzaldehyde	2767.3, 3065.3, 1825.2, 2742.2, 2438.5, 2040.3, 2628.5, 1781.9, 2182, 2353.7	8063, 9573, 6973, 9735, 9872, 8297, 6921, 5769, 7243, 6922
*p*-Cymene	2912, 3179.1, 1282.4, 922.71, 2144.4, 2944.7, 2395.1, 1788.6, 798.32, 3451	5474, 4690, 4735, 6648, 6625, 6872, 5149, 6556, 4925, 6969
Decanal	1628.5, 2845.4, 3023.8, 2442.4, 2000.7, 3578.3, 1402.9, 2102.9, 1032.6, 2034.5	6840, 6976, 7093, 8287, 6894, 8213, 8827, 7132, 9380, 9647
Dodecane	1978.6, 1133.9, 2229.3, 1804, 780.96, 3428.8, 2762.5, 3098.1, 2912.9, 3171.3	4738, 6698, 4515, 6827, 4879, 4920, 4467, 5109, 4854, 6571
Farnesene	1805, 1626.6, 2291.9, 3316, 2772.1, 896.67, 3046, 1563.9, 1891.8, 2132.8	7695, 7415, 11,224, 8069, 6290, 6104, 4926, 8368, 8155, 4879
Hexyl acetate	2279.4, 1678.7, 647.9, 1694.1, 2796.2, 1199.4, 2758.6, 2957.3, 3144.3, 2212.9	6938, 4773, 10,229, 6799, 6817, 5956, 6910, 6242, 6356, 7135
Limonene	3488.6, 2373.9, 2700.8, 3199.3, 2363.3, 2664.1, 3562.8, 3236.9, 3081.7, 2501.2	7616, 6810, 8867, 6430, 4849, 7000, 6809, 5188, 6469, 6950
Menthol	2074, 2832.9, 268.95, 3674.7, 2774.1, 123.34, 1687.4, 1660.4, 2960.2, 3104.8	5290, 7403, 7602, 8838, 8596, 9014, 8958, 5396, 8937, 7774
Menthone	2439.5, 3187.7, 2306.4, 2635.2, 3515.6, 2284.2, 2467.4, 3397, 2138.6, 632.47	11,963, 10,033, 7719, 6021, 11,606, 10,410, 8547, 5971, 5965, 11,433
Myrcene	1856.1, 2736.5, 1438.6, 2858, 2360.4, 591.01, 1465.6, 1011.4, 1926.5, 3319.8	6726, 5206, 6695, 6766, 6952, 4625, 6934, 7183, 6688, 6158
Nonanal	1662.3, 3493.4, 2831.9, 3037.3, 1762.6, 2920.6, 2955.3, 1476.2, 3200.3, 1408.7	5802, 6821, 4869, 6957, 8568, 6520, 6627, 6988, 5466, 6836
Pentanal	2836.7, 2381.6, 1941.9, 2378.7, 2419.2, 3441.3, 3491.5, 671.04, 2819.4, 2348.8	6710, 4433, 4821, 7986, 6884, 6759, 4408, 4522, 6752, 6251
Pulegone	2007.5, 2150.2, 2609.2, 2122.2, 3506.9, 2345, 2342.1, 2119.3, 2292.9, 1768.4	5121, 11,821, 6989, 5247, 5853, 5930, 5143, 11,332, 5033, 10,154
Sabinene	1857.1, 3690.1, 3631.3, 3674.7, 3662.1, 1927.5, 1788.6, 2533, 1547.5, 3586	5049, 11,397, 4707, 4541, 5937, 4940, 5767, 8698, 5996, 4113

**Table 6:** PLSR for modeling EWs for Raman and NIR data.

Technology	RAMAN							NIR						
	LV	R^2^	RMSE	RPD	R^2^	RMSE	RPD	LV	R^2^	RMSE	RPD	R^2^	RMSE	RPD
HRY	1	0.8559	1.835	3.62	0.8449	1.9472	3.38	2	0.8109	2.1025	2.73	0.787	2.2822	2.30
SG	1	0.9406	4.0641	3.98	0.9365	4.2976	3.75	3	0.9788	2.4271	3.30	0.9743	2.7317	2.85
1-Octen-3-ol	2	0.8197	0.2009	3.47	0.7511	0.2415	3.01	2	0.9615	0.0928	3.24	0.954	0.1037	2.79
Acetophenone	2	0.7825	0.0323	3.31	0.6977	0.039	2.79	2	0.8798	0.024	2.97	0.8559	0.0269	2.50
Benzaldehyde	2	0.8975	0.086	3.80	0.8629	0.1017	3.46	2	0.9488	0.0607	3.20	0.9404	0.0671	2.75
*p*-Cymene	1	0.9336	0.08392	3.95	0.9303	0.8795	3.73	3	0.8793	1.132	2.97	0.8618	1.2388	2.52
Decanal	2	0.8688	0.1872	3.68	0.8413	0.2106	3.37	2	0.8915	0.1703	3.01	0.8762	0.186	2.56
Dodecane	1	0.6799	5.8066	2.88	0.6718	6.0133	2.69	2	0.6296	6.2458	2.12	0.5724	6.8631	1.67
Farnesene	1	0.0644	0.0458	0.27	0.01785	0.048	0.07	1	0.0046	0.0473	0.02	NA	0.0514	0.03
Hexyl acetate	2	0.8786	0.9581	3.72	0.859	1.0562	3.44	1	0.8239	1.1541	2.78	0.8164	1.2053	2.39
Limonene	1	0.8633	0.5463	3.65	0.8504	0.5845	3.41	3	0.9199	0.418	3.10	0.9085	0.4572	2.66
Menthol	4	0.4156	0.0988	1.76	0.1073	0.1249	0.43	1	0.0469	0.1261	0.16	0.0119	0.1314	0.03
Menthone	1	0.0501	0.0143	0.21	NA	0.0152	0.23	6	0.3272	0.012	1.10	0.1295	0.014	0.38
Myrcene	2	0.7913	0.09055	3.35	0.7254	0.1062	2.90	2	0.8564	0.0751	2.89	0.8348	0.0823	2.44
Nonanal	2	0.9216	0.5641	3.90	0.9042	0.6375	3.62	2	0.9351	0.5132	3.15	0.9223	0.5743	2.70
Pentanal	1	0.9184	0.059	3.89	0.9133	0.0622	3.66	2	0.9454	0.0482	3.19	0.9337	0.0544	2.73
Pulegone	1	0.3871	0.01673	1.64	0.34	0.0177	1.36	3	0.4491	0.0158	1.51	0.3365	0.0178	0.98
Sabinene	1	0.5701	0.0268	2.41	0.5248	0.0289	2.10	4	0.9015	0.0128	3.04	0.865	0.0154	2.53

**Table 7:** ANN for modeling EWs for Raman and NIR data.

Technology	RAMAN									NIR								
	Training			Validation			Test			Training			Validation			Test		
	R^2^	RMSE	RPD	R^2^	RMSE	RPD	R^2^	RMSE	RPD	R^2^	RMSE	RPD	R^2^	RMSE	RPD	R^2^	RMSE	RPD
HRY	0.93	2.1436	3.93	0.82	1.3218	3.84	0.94	2.6747	3.66	0.99	0.58	5.37	0.98	0.77	4.90	0.97	1.09	4.21
SG	0.95	5.7048	4.03	0.83	7.8689	3.88	0.89	5.7450	3.91	0.99	1.52	5.58	0.99	2.28	4.93	0.97	3.31	4.41
1-Octen-3-ol	0.76	0.2265	3.71	0.75	0.3089	3.53	0.72	0.2706	3.59	1.00	0.02	5.80	0.97	0.08	4.86	0.97	0.11	4.23
Acetophenone	0.86	0.0332	3.66	0.73	0.0200	3.43	0.81	0.0224	2.15	0.98	0.01	5.33	0.97	0.01	4.85	0.79	0.06	2.22
Benzaldehyde	0.92	0.0980	3.89	0.85	0.1192	3.67	0.96	0.0566	3.72	0.99	0.03	5.36	0.97	0.07	4.82	0.96	0.05	4.19
*p*-Cymene	1.00	0.0000	∞	0.98	0.4407	4.01	0.92	0.9635	3.58	1.00	0.05	5.41	0.92	1.10	4.58	0.99	0.33	4.31
Decanal	0.88	0.2594	3.44	0.84	0.2304	3.33	0.91	0.2100	3.52	1.00	0.02	5.40	0.99	0.05	4.93	0.91	0.17	3.98
Dodecane	1.00	0.0000	∞	0.84	3.2998	2.31	0.66	7.2673	1.56	0.95	2.36	5.15	0.83	5.88	4.16	0.53	7.33	2.31
Farnesene	0.24	0.0458	1.00	0.09	0.0374	0.41	0.01	0.0678	0.02	0.34	0.03	1.13	0.13	0.05	0.63	0.36	0.06	1.06
Hexyl acetate	1.00	0.0520	4.24	0.97	0.8691	4.55	0.96	0.7977	3.74	0.99	0.43	5.38	0.95	0.56	4.75	0.99	0.63	4.33
Limonene	1.00	0.0000	∞	0.96	0.7690	4.49	0.84	0.6178	3.27	0.99	0.24	5.33	0.97	0.29	4.85	0.99	0.20	4.33
Menthol	0.61	0.0678	2.60	0.17	0.1476	0.81	0.07	0.1761	0.28	0.46	0.10	1.12	0.29	0.09	1.06	0.05	0.14	0.22
Menthone	0.03	0.0140	0.14	0.32	0.0107	1.02	0.04	0.0207	0.14	0.39	0.01	1.10	0.58	0.01	1.31	0.15	0.02	0.66
Myrcene	0.99	0.0173	4.21	0.88	0.1034	4.12	0.67	0.1655	1.61	0.97	0.04	5.27	0.99	0.03	4.92	0.86	0.06	2.73
Nonanal	1.00	0.0000	∞	0.96	0.4512	4.51	0.87	0.5860	2.37	0.97	0.43	5.23	0.95	0.42	4.72	0.99	0.39	4.30
Pentanal	0.98	0.0500	4.15	0.97	0.0332	4.52	0.93	0.0906	3.63	1.00	0.01	5.40	1.00	0.02	4.97	1.00	0.02	4.34
Pulegone	0.43	0.0174	1.22	0.73	0.0102	2.13	0.69	0.0128	1.98	0.71	0.01	1.86	0.68	0.01	1.41	0.53	0.01	1.31
Sabinene	0.81	0.0201	2.11	0.58	0.0249	1.23	0.68	0.0297	1.63	0.95	0.01	5.16	0.94	0.01	4.89	0.96	0.04	4.16

**Table 8:** Description of various soaking methods.

Soaking Method	Temperature (°C)	Soaking Time (min)	Steaming Time (min)
First	60	15, 30, 60, 120, 240	0
	65	15, 30, 50, 95, 180	
	70	15, 25, 40, 70, 120	
Second	Temperature (°C)	Drying temperature(°C)	Drying time (min)
	60	50	10, 40, 70, 95, 120
		60	10, 35, 60, 75, 90
		70	10, 20, 30, 40, 50
	65	50	10, 40, 70, 95, 120
		60	10, 35, 60, 75, 90
		70	10, 20, 30, 40, 50
	70	50	10, 40, 70, 95, 120
		60	10, 35, 60, 75, 90
		70	10, 20, 30, 40, 50

## Data Availability

Data are contained within the article.

## Abbreviations

SG	Starch Gelatinization
HRY	Head Rice Yield
PLSR	Partial Least Squares Regression
PCA	Principal Component Analysis
CV	Cross-Validation
P	Prediction
LA	Learning Automata
SPME	Solid Phase Microextraction
RMSE	Root Mean Square Error
R^2^	R-squared
RPD	Relative Percent Deviation
LV	Latent Variable
C	Calibration
EWs	Effective Wavelengths
SVM	Support Vector Machine
